# Forensic integrity of blood alcohol sampling in the emergency department, Part I: Epidemiology and factors affecting turnaround time

**DOI:** 10.11613/BM.2026.030703

**Published:** 2026-08-10

**Authors:** Maria Sapio, Anna Criniti, Simona Giglio, Gioacchino Galardo, Orietta Gandini, Antonio Angeloni, Cristiano Ialongo

**Affiliations:** 1 Department of Clinical Pathology, University Hospital Policlinico Umberto I, Rome, Italy; 2 Department of Experimental Medicine, Sapienza University of Rome, Rome, Italy; 3 Department of Medical and Cardiovascular Sciences, Sapienza University of Rome, Italy; 4 Department of Molecular Medicine, Sapienza University of Rome, Rome, Italy

**Keywords:** blood alcohol content, hospital emergency service, preanalytical phase, substance abuse detection, retrospective study

## Abstract

**Introduction:**

Drink-driving remains a leading global cause of road traffic fatalities. In Italy, the determination of blood alcohol concentration (BAC) in injured drivers relies on coordination between law enforcement and emergency departments (ED) to ensure legally admissible evidence. Because ethanol undergoes continuous metabolic decay, any delay in blood sampling can lead to underestimated values or “pharmacokinetic false negatives”.

**Materials and methods:**

A retrospective study was conducted throughout 2025 on 115 drivers admitted to a major university hospital following road accidents. Preanalytical turnaround time (TAT), categorized into intra-ED and extra-ED intervals, was assessed against variables like clinical severity (triage code), temporal patterns (duty shifts/weekends), and licensure status. Serum alcohol concentration (SAC) that is used to screen BAC was measured with the enzymatic methods.

**Results:**

The population was predominantly male (77.4%) with a median age of 36 years. While the median intra-ED TAT was 139 minutes and remained stable across various clinical and temporal factors, the overall preanalytical TAT (from accident to sampling) reached a median of 204 minutes. The only statistically significant association concerned SAC at ED admission with ED duty shift (P = 0.045), and with day of the week (P = 0.035).

**Conclusions:**

The observed TAT appears insensitive to environmental stressors, potentially indicating a “degraded” baseline performance. This study emphasizes that objective quality goals for TAT cannot rely solely on administrative averages. Instead, they must be grounded in a quantitative relationship between sampling delay and metabolic decay using pharmacokinetic models to mitigate the risk of forensic evidence loss.

## Graphical abstract

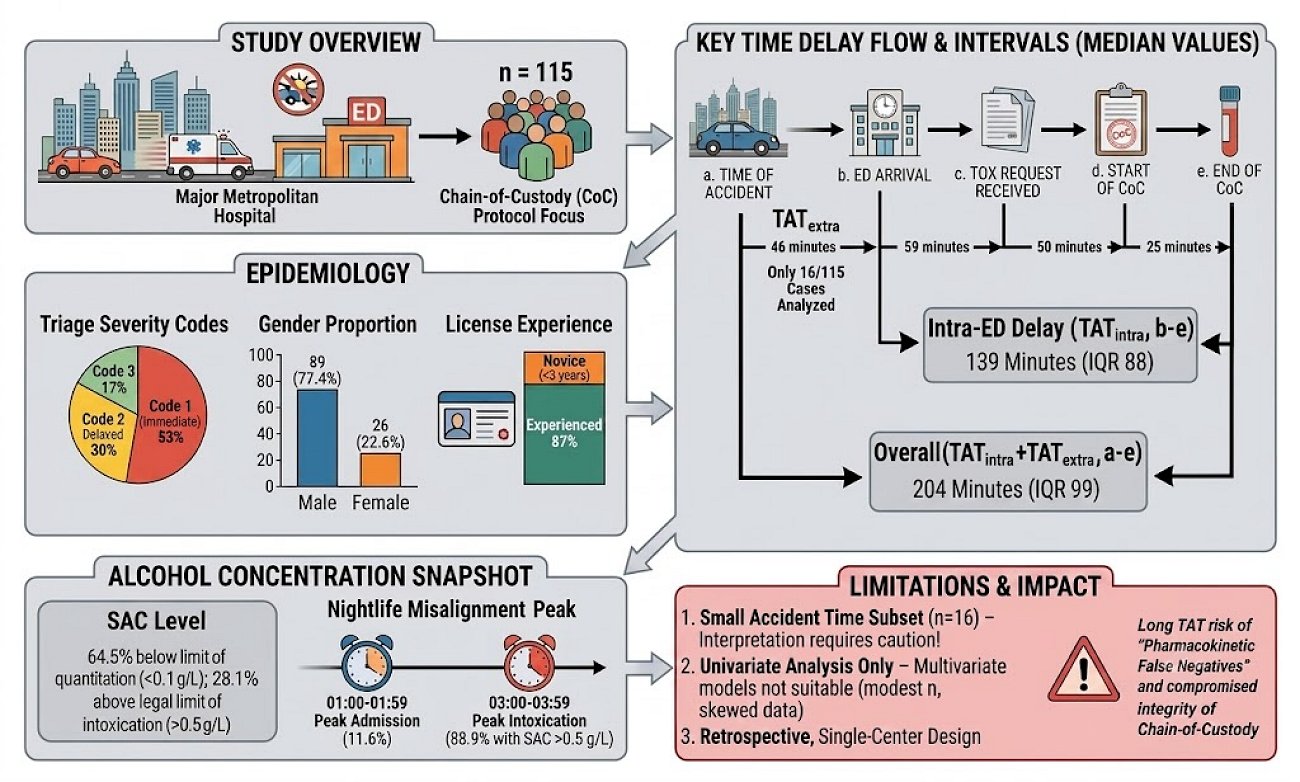

## Highlights

• Median intra- emergency departments (ED) turnaround time for forensic alcohol sampling is 139 minutes

• The primary bottleneck is the 57-minute delay in requesting toxicological tests

• Intra-ED sampling delays are independent of triage priority and duty shifts

• Total preanalytical delay from accident to sampling reaches a median of 204 min

• Sampling delays remain stable regardless of hospital workload or time of day

## Introduction

Drink-driving is one of the leading causes of death and serious injury worldwide, affecting drivers, passengers, and pedestrians alike. It is well documented that alcohol impairs the cognitive and motor functions essential for safe driving, leading to a markedly increased risk of road traffic crashes even at relatively low blood alcohol concentration (BAC) (*1*, *2*). Globally, it is estimated that 5% to 35% of all road traffic fatalities are attributable to driving under the influence of alcohol, with substantial regional variability related to legislation, cultural factors, and the enforcement of traffic safety laws (*3*, *4*).

In Europe, approximately 25% of all road traffic deaths are alcohol-related (*5*). In response, the European Union (EU) has identified drink-driving as a key target for prevention and enforcement, setting since 2011 the objective of reducing road fatalities by 50% (*6*). These policy orientations have reinforced the need for effective legal and clinical procedures to ensure timely and reliable assessment of alcohol impairment.

In Italy, the enforcement of drink-driving laws in traffic accidents resulting in injured drivers relies on close coordination among law enforcement agencies, emergency medical services, and hospital emergency departments (EDs). This integrated framework is designed to ensure both timely and appropriate medical care and the legally admissible determination of BAC as close as possible to the time of the incident. Such timing is crucial because, due to the pharmacokinetics of ethanol, delayed blood sampling may lead to falsely negative or underestimated BAC values (*7*).

While playing a pivotal role in this process, the ED is frequently burdened by high workloads, time pressure, and overcrowding, factors that can compromise the timeliness of blood sampling. Additionally, the legal requirements for BAC testing necessitate a rigorous and time-consuming chain of custody (CoC) protocol. In cases involving severe offenses, such as ‘vehicular homicide’, blood testing results must be provided strictly within 48 hours of the blood draw. Consequently, the burden of the CoC process extends beyond the ED to the hospital’s clinical laboratory. To expedite the overall turnaround time (TAT), the CoC workflow may involve an initial screening phase of serum alcohol concentration (SAC) measured with the enzymatic alcohol dehydrogenase (ADH) reagent on clinical chemistry analyzers (*8*). Thereafter, confirmatory analysis *via* headspace gas-chromatography (HS-GC) is performed, sometimes directly within the same laboratory setting.

Against this background, this research has been developed as two-part study. The present article (Part I) aims to provide epidemiological data on drink-driving cases at a major metropolitan university hospital, and to identify the factors influencing timely blood sampling for SAC analysis within the CoC framework. Part II of this study (published separately) assess TAT for SAC within CoC in the ED to establish evidence-based quality goals of performance (*9*).

## Materials and methods

### Study population and data collection

Data were collected retrospectively from January 1st to December 31st, 2025 (ethical committee approval on laboratory data retrospective analysis nr.0518/2024). The study included drivers involved in road accidents and admitted to the ED of Azienda Ospedaliero-Universitaria Policlinico Umberto I of Rome, for whom a forensic toxicological determination of alcohol concentration was requested in compliance with the provisions of the Italian Highway Code (*Codice della Strada*). Since the Italian Highway Code defines legal limits in terms of BAC, but an initial screening phase of SAC measured with the ADH method is performed for CoC, all analytical measurements and data stratifications within this study are reported strictly as SAC.

Newly licensed drivers and professional drivers, for whom Italian law mandates a zero-tolerance policy for the use of any psychotropic substances, were also included. All the specimens were collected and processed in the CoC.

### Demographic variables

Descriptive epidemiological analyses included patient age at the time of ED admission and sex, and driving status, with particular attention to newly licensed drivers (< 3 years *vs*. ≥ 3 years of licensure), in accordance with the Italian Highway Code.

### Preanalytical turnaround time and influencing variables

**Figure 1:**
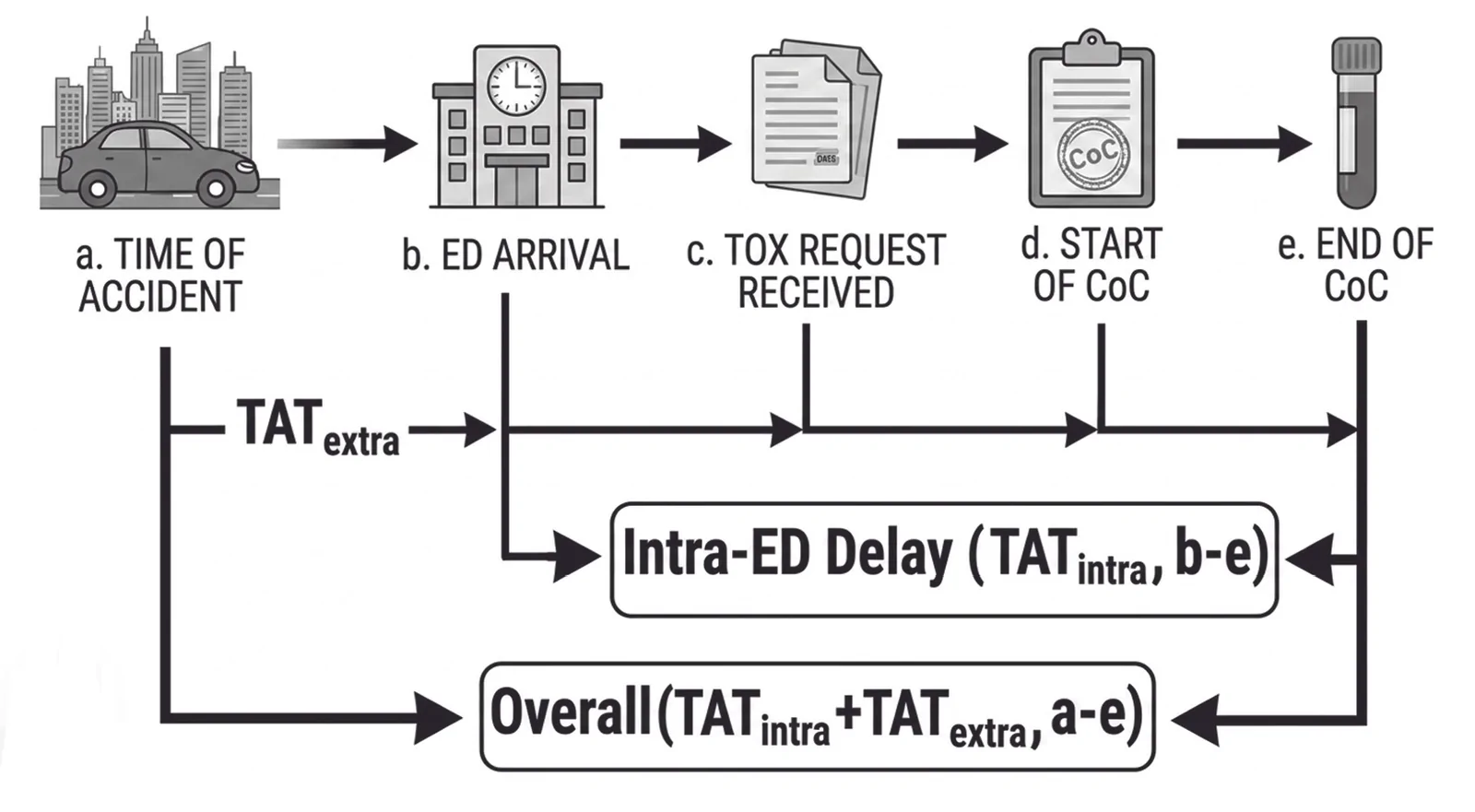
Schematic flowchart of the preanalytical turnaround time (TAT) workflow and intervals. For the assessment of preanalytical TAT, the following definitive time points were considered: (a) presumed time of the accident; (b) time of emergency department (ED) admission; (c) time of receipt of the toxicological testing request issued by prosecutorial or law enforcement authorities; and (d) the start and (e) end times of biological sample collection within the chain of custody (CoC).

For the assessment of preanalytical TAT, the following time points were considered: (a) presumed time of the accident; (b) time of ED admission; (c) time of receipt of the toxicological testing request issued by prosecutorial or law enforcement authorities; and (d) the start and (e) end times of sample collection within the chain of custody. Accordingly, the following intra-ED TAT intervals were calculated: b–c, c–d, d–e, as well as the total intra-ED preanalytical TAT (TAT_intra_) was defined b–e. The extra-ED preanalytical TAT (TAT_extra_) was defined as a–b; therefore, the overall preanalytical TAT (TAT_intra_+ TAT_extra_) was defined as a–e. The time points are represented in Figure 1.

Variables potentially influencing TAT included the severity of the patient’s clinical condition at admission, classified according to the triage numerical/color code (1–2–3; red–yellow–green); temporal patterns of ED arrival time according to duty shifts, categorized as daytime (08:00–14:00), afternoon (14:00–20:00), or nighttime (20:00–08:00); and the day of the week (weekday *vs.* weekend/holiday).

### Toxicological analysis

Serum alcohol concentration testing was performed on Ilab Taurus chemistry analyzer (Instrumentation Laboratory S.p.A., Milan, Italy). Where appropriate, SAC values were categorized as < 0.1 g/L (limit of quantitation of the ADH method), 0.1-0.5 g/L (below the legal limit of intoxication), 0.5-0.8 g/L (first grade of intoxication above the legal limit), 0.8-1.5 g/L (second grade) and > 1.5 g/L (third grade).

### Statistical analysis

Outliers in the TAT were identified using the available CoC information and were then excluded from the analysis. All data were preliminarily assessed for normality using the Anderson-Darling test. In the absence of a normal distribution, continuous variables were reported as the median and interquartile range (IQR). Differences in medians were evaluated using either the Mann-Whitney test or the Kruskal-Wallis test, depending on the number of groups compared, while associations between categorical variables were assessed using Fisher’s or Fisher-Freeman-Halton’s exact test. All analyses were performed on the total sample size, unless otherwise specified. Confidence intervals on percentiles were computed using the bias-corrected accelerated (BCa) bootstrap. Statistical significance was set at P < 0.05. All statistical analyses were conducted using Minitab software (version 22.1; Minitab, LLC, State College, USA).

## Results

### Epidemiological data

The study population consisted of 121 individuals, 115 of whom were included in the final analysis as they were associated with known cases of prolonged delay (*e.g.,* incorrect name in the first toxicological analysis request which caused a resubmission of the request). Data are summarized in Table 1. Age distribution did not differ significantly between the genders (P = 0.970). No professional drivers were present.

**Table 1:** Summary data of the case series demographics and driving experience

**Variable**	**Males (N = 89)**	**Females (N = 26)**	**Total (N = 115)**
Median age (IQR)	36.2 (28.3)	35.6 (27.6)	36.2 (26.6)
License < 3 years, N (%)	11 (12.4%)	4 (15.4%)	15 (13.0%)
License > 3 years, N(%)	78 (87.6%)	22 (84.6%)	100 (87.0%)

**Figure 2:**
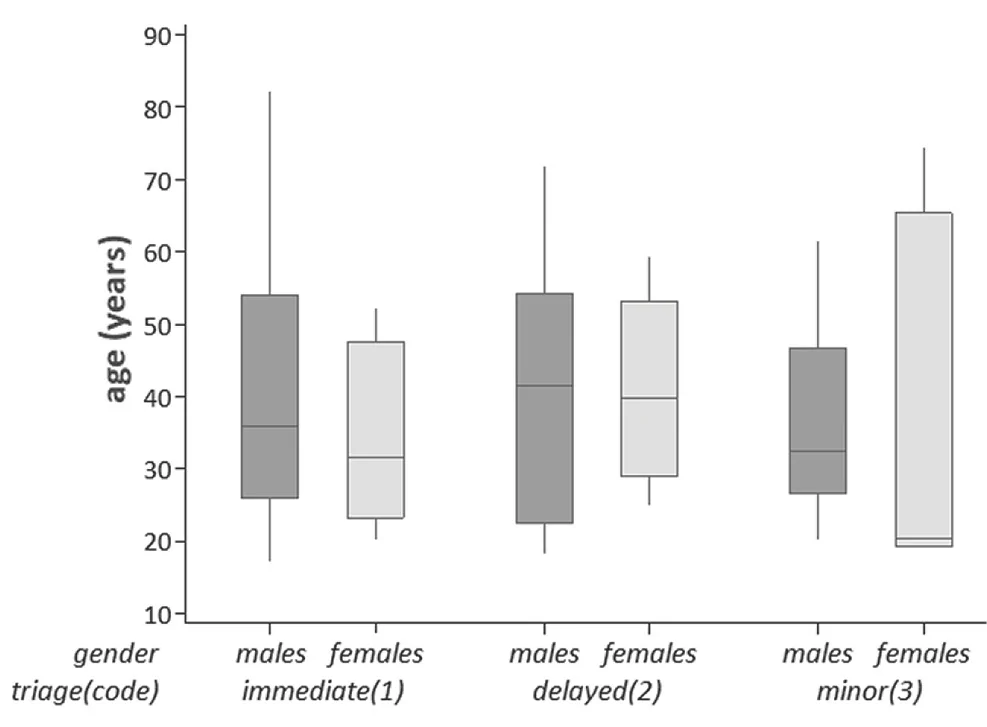
Box-plot representing the age distribution of drivers (N = 115) admitted to the emergency department for forensic toxicological analysis following road traffic accidents, stratified by gender and clinical severity (triage-assigned code).

With regard to trauma severity, 61 individuals (53%; 50 males and 11 females) were classified as triage code 1 (immediate), 34 (29.6%; 24 males and 10 females) as triage code 2 (delayed), and 20 (17.4%; 15 males and 5 females) as triage code 3 (minor). The distribution of patient age stratified by sex and triage code is shown in Figure 2.

Regarding temporal patterns of ED admissions, 10.7% occurred during the daytime shift (08:00–14:00), 19.8% during the afternoon shift (14:00–20:00), and 69.4% during the nighttime shift (20:00–08:00). Overall, 62.8% of admissions occurred on weekdays (Monday–Friday), while 37.2% occurred on weekends or public holidays.

**Figure 3:**
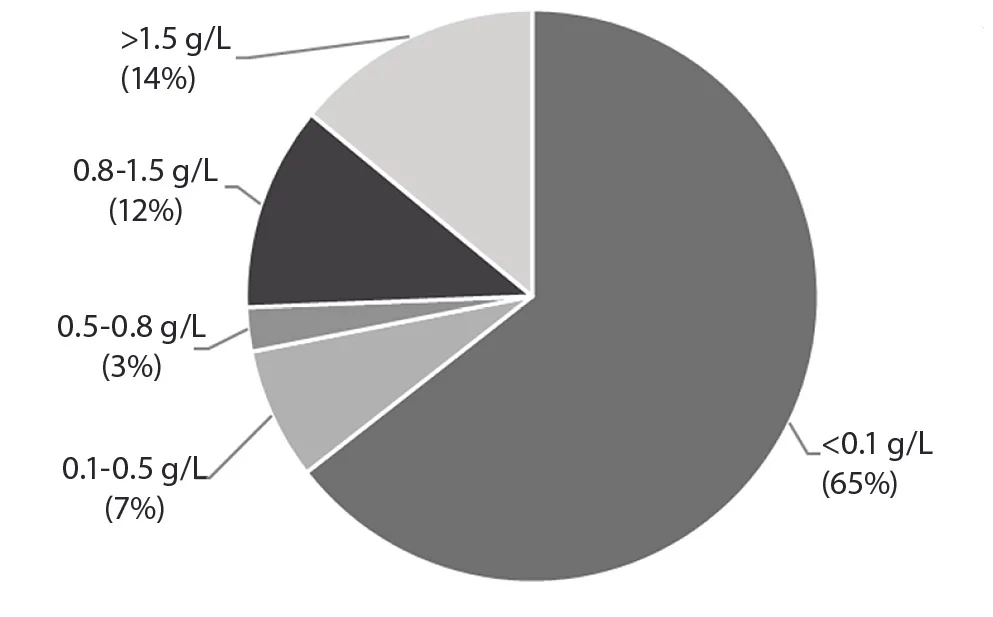
Distribution of serum alcohol concentrations (SAC) among drivers (N = 115) admitted to the emergency department for forensic toxicological analysis following road traffic accidents. The limit of quantitation for the automated enzymatic method was 0.1 g/L. In accordance with the Italian Highway Code, 0.5 g/L represents the legal limit of intoxication (LLI), while 0.8 g/L and 1.5 g/L represent the second and third grades of intoxication, respectively.

Overall, 28.1% of subjects presented with a SAC > 0.5 g/L, corresponding to the legal limit of intoxication (LLI) in Italy for non-professional drivers holding a driving license for more than 3 years. Within this subgroup, the median SAC was 1.48 g/L (IQR = 0.5 g/L). By contrast, subjects with SAC < 0.1 g/L (limit of quantitation of the ADH method) were 64.5%. The distribution of SAC is summarized in Figure 3. Since almost two-third of the SAC values were below the limit of quantitation, statistical tests were performed on the data categorized as shown in Figure 3. The association between SAC at ED admission and study variables is summarized in Table 2.

**Table 2:** Association between serum alcohol concentration (SAC) at emergency department (ED) admission, trauma severity and age of driving licence with demographic/temporal variables

**Independent Variable**	**Dependent Variable**	**P-value (Fisher-Freeman-Halton exact test)**
SAC at ED admission	Driver sex	0.794
	Age of driving license	0.949
	Trauma severity (triage code)	0.340
	ED duty shift	0.045
	Day of the week	0.035*
Trauma severity (Triage Code)	Driver sex	0.567
	ED duty shift	0.329
	Day of the week	0.634
Age of driving license	Trauma Severity	0.536
	ED duty shift	0.569
	Day of the week	0.138
*Computed with Kruskal-Wallis test.		

**Figure 4:**
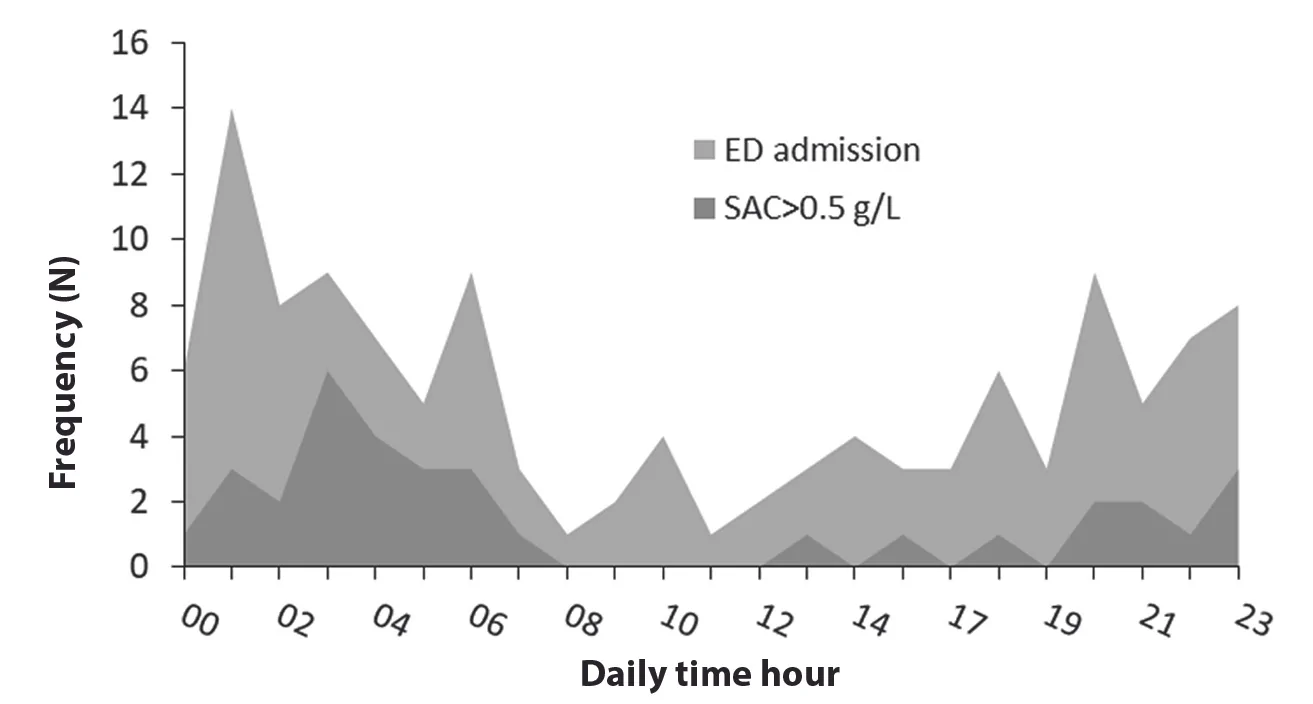
Hourly distribution of serum alcohol concentrations (SAC) and cases exceeding the Italian legal limit of alcohol intoxication (SAC > 0.5 g/L) among drivers (N = 115) admitted to the emergency department following road traffic accidents for forensic toxicological analysis.

Independently of the day of the week, the peak of ED admissions (11.6%) occurred during the 01:00–01:59 time interval, within which 28.6% of patients had a SAC > 0.5 g/L. Conversely, during the 03:00–03:59 interval, accounting for 7.4% of total admissions, the proportion of patients with SAC > 0.5 g/L reached 88.9%. Detailed hourly distributions of ED admissions and cases with SAC > 0.5 g/L are shown in Figure 4. The association between severity of trauma according to the triage-assigned code, license experience and study variables is summarized in Table 2.

### Turnaround time data

The median TAT_intra_ was 139 minutes (IQR = 88 minutes). The stratification of TAT values for the individual intervals within the preanalytical phase is reported in Table 3. In 16 cases for which the presumed time of the accident could be retrieved from the toxicological testing request documentation, the median TAT_extra_ was 46 minutes (IQR = 19 minutes).

**Table 3:** Stratification of turnaround time (TAT) of the intra- and extra-emergency department (ED) preanalytical phases for sample collection within the chain of custody

**TAT interval**	**Median**	**IQR**	**95th percentile**	**95%CI**
**Intra-ED TAT**	Minutes			
(b–c) From ED admission to toxicological tests request	59	57	149	120-159
(c–d) From toxicological tests request to start of the chain of custody	50	45	168	144-228
(d–e) From start to end of the chain of custody	25	26	71	60-111
(b–e) **Total intra-ED preanalytical TAT (TAT_intra_)**	139	88	294	271-320
Extra-ED*	Minutes			
(a–b) From time of accident to ED admission **(TAT_extra_)**	46	21	86	61-88
(a–e) **Overall pre-analytical TAT (TAT_intra_ + TAT_extra_)**	204	99	420	280-510
*Computed in 16 out of 121 cases. CI – confidence interval.				

**Figure 5:**
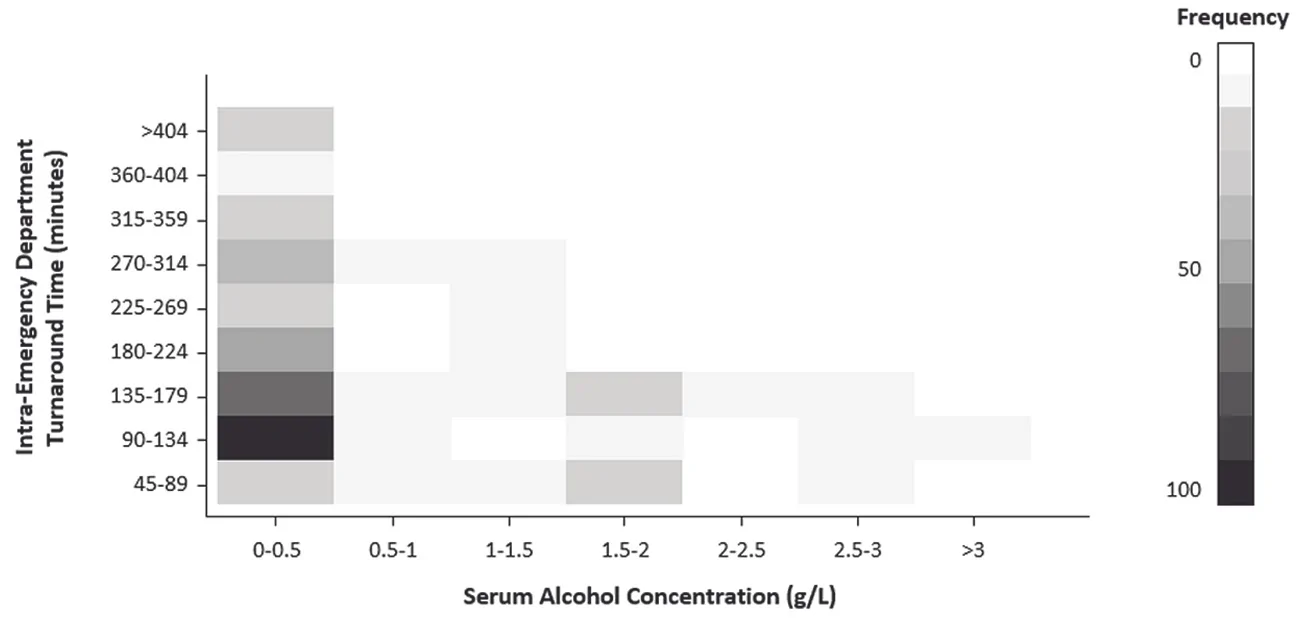
Heatmap of intra-emergency department turnaround time (TAT_intra_) according to serum alcohol concentration (SAC) among drivers (N = 115) admitted for forensic toxicological analsis following road traffic accidents.

With respect to influencing variables, the median TAT_intra_ did not differ significantly according to trauma severity as assessed by triage code (P = 0.372), ED duty shift (P = 0.519), or day of the week (P = 0.981). Table 4 summarizes the results of the statistical analyses performed on the individual TAT_intra_ intervals. Notably, TAT and SAC were not significantly associated (P = 0.934). The distribution of TAT_intra_ according to the level of SAC is shown in Figure 5.

**Table 4:** Statistical significance of factors influencing intra-emergency department (ED) turnaround time (TAT) intervals for sample collection within the chain of custody, assessed using the Kruskal-Wallis test

	**Factors**		
**TAT interval**	**Severity of trauma**	**ED duty shift**	**Day of the week**
Intra-ED TAT (TAT_intra_)	P-value		
(b-c) From ED admission to toxicological test request	0.541	0.927	0.694
(c-d) From toxicological test request to start of the chain of custody	0.173	0.455	0.094
(d-e) From start to end of the chain of custody	0.814	0.645	0.957

## Discussion

This single-center one-year retrospective evaluation of the epidemiology of drink-driving cases admitted to the ED of a large metropolitan hospital shows an epidemiological framework made up predominantly composed by males, with a ratio of nearly 4:1 relative to females and a median age of approximately 40 years in both groups. This gender gap aligns with national and global statistics, which consistently identify middle-aged males as being significantly more involved in alcohol-related road accidents (*10*, *11*).

Cases of high clinical severity (triage code 1) accounted for half of the recorded instances. Interestingly, novice drivers (licensed < 3 years), who represented approximately 15% of the total, did not show an association with increased trauma severity. Unexpectedly, trauma severity did not vary significantly with SAC. This finding may be due either to the unaccounted consumption of other substances of abuse that impair driving performance, or by the large prevalence of cases with SAC below the limit of quantitation of the ADH method (*12*).

The temporal distribution of events shows that two-thirds of admissions occurred on weekends or holidays, and two-thirds during the night shift, reflecting the prevalence of recreational ethanol consumption (*13*). Notably, the level of SAC peaked during the night and in the weekends or holidays. A peculiar misalignment exists between the peak of total driver admissions (01:00–01:59) and the peak of drivers exceeding the legal limit of 0.5 g/L (03:00–03:59). It is hypothesized that this reflects a distinct pattern of alcohol consumption: the 03:00–03:59 peak likely involves accidents occurring in recreational contexts where alcohol abuse is a central feature and drinking is concentrated within a short interval (*14*, *15*). These individuals face higher alcohol levels and a hazardous interaction between psychotropic effects, fatigue, and the high cognitive load required by night driving.

As such, the ED’s peak forensic activity coincides with its most challenging operational hours (*16*). Nevertheless, the median TAT_intra_ was influenced neither by the severity of injuries nor by the specific time slot in which the CoC was executed. This result contrasts with recent evidence from a large cohort study (N = 8923), where drivers admitted during the night shift and patients with the most severe injuries experienced longer TATs (*17*).

High-severity traumas requiring urgent clinical intervention typically lead to significant delays in forensic sampling due to the prioritization of medical stabilization (*17*). In contrast, our setting demonstrated exceptional stability, the interpretation of which is puzzling. On one hand, this could be viewed as a positive outcome, demonstrating the ED medical team’s proficiency and standardized mastery of CoC protocols that ensures “forensic equity” for all drivers. On the other hand, it allows for a more critical interpretation: such stability might indicate a “degraded” baseline TAT, one so high that it remains insensitive to additional environmental or clinical stressors.

Quantitatively, literature indicates that an average preanalytical TAT of 1.8 hours (108 minutes) is expected, with approximately 44% of forensic samples collected within 2 hours (roughly corresponding to a median of slightly more than 120 minutes) of the accident and 81.3% within 3 hours (*17*, *18*). Recalling that the TAT has usually a skewed distribution which makes the average useless, our median TAT_intra_ of 139 minutes was reasonably consistent with the state of the art. This is especially relevant considering that a median of 57 minutes was consumed by the waiting for the toxicological test request alone, a process which can be easily improved. However, accounting for the TAT_extra_ (from the presumed time of the accident to ED admission) increased the median TAT to 204 minutes (approximately 3.4 hours), which conversely appears unacceptably long.

Regardless of the underlying cause (*e.g.,* ED overcrowding, staffing shortages, or communication difficulties with law enforcement), an objective judgment on whether the observed TAT is truly degraded or forensically “safe” requires evidence-based criteria. Quality goals must be grounded in a quantitative relationship between sampling delay and the potential loss of forensic evidence due to metabolic decay, a risk we may term “pharmacokinetic false negatives.”

While this study provides valuable real-world insight into the operational workflow of forensic blood alcohol sampling within a major metropolitan emergency department, several limitations must be acknowledged.

First, a key finding is the substantial overall preanalytical delay between the accident and blood sampling, which yielded a median time of 204 minutes. However, because the exact time of the road accident was missing or unrecorded in the vast majority of law enforcement requests, this specific interval could only be analyzed for a small subset of the study population (N = 16 out of 121 cases). This small sub-cohort may not be fully representative of the entire study population, and the calculated TAT_extra_ should be interpreted with caution.

Second, the statistical evaluations rely exclusively on univariate analyses. Given the complex environment of an overcrowded emergency department, multiple clinical, demographic, and temporal variables likely interact dynamically (*e.g*., triage severity, night shifts, and weekend staffing). A multivariable regression model could provide stronger evidence regarding independent predictors of prolonged TAT. However, due to the modest final sample size N = 115) and the heavily skewed distributions of categorical data (*e.g*., nearly 70% of admissions occurring during night shifts), a multivariable approach risked overfitting the statistical models. Therefore, univariate tests were prioritized as a more reliable baseline analysis.

Finally, the study is limited by its retrospective, single-center design, which may reflect local operational habits or institutional arrangements that restrict direct generalizability to other clinical settings. Additionally, the analysis did not screen for the potential co-ingestion of illicit psychotropic substances or prescription medications, which frequently co-occur in traffic accidents and can heavily influence clinical triage prioritization.

In conclusion, establishing an evidence-based extra-analytical quality criterion requires a probabilistic and statistical framework since a direct experimental approach is ethically and practically unfeasible. Specifically, the known pharmacokinetics of ethanol provides a quantitative model suitable for determining performance specifications *via* Monte Carlo numerical simulations. Due to the complexity of this method and its significant implications, this simulation-based approach is addressed separately in the Part II of this work (*9*).

## Data Availability

The data generated and analyzed in the presented study are available from the corresponding author on request.
